# Upregulation of Autophagy-Related Gene-5 (ATG-5) Is Associated with Chemoresistance in Human Gastric Cancer

**DOI:** 10.1371/journal.pone.0110293

**Published:** 2014-10-17

**Authors:** Jie Ge, Zihua Chen, Jin Huang, Jinxiang Chen, Weijie Yuan, Zhenghao Deng, Zhikang Chen

**Affiliations:** 1 Department of Gastrointestinal Surgery, Xiangya Hospital, Central South University, Changsha, Hunan, P. R. China; 2 Department of Oncology, Xiangya Hospital, Central South University, Changsha, Hunan, P. R. China; 3 Department of Pathology, Xiangya Hospital, Central South University, Changsha, Hunan, P. R. China; University of Nebraska Medical Center, United States of America

## Abstract

Autophagy-related gene-5 (ATG-5) is one of the key regulators of autophagic cell death. It has been widely regarded as a protective molecular mechanism for tumor cells during the course of chemotherapy. In the present study, we investigated the expression pattern of ATG-5 and multidrug resistance-associated protein-1 (MRP-1) in 135 gastric cancers (GC) patients who were treated with epirubicin, cisplatin and 5-FU adjuvant chemotherapy (ECF) following surgical resection and explored their potential clinical significance. We found that both ATG-5 (77.78%) and MRP-1 (79.26%) were highly expressed in GC patients. ATG-5 expression was significantly associated with depth of wall invasion, TNM stages and distant metastasis of GC (P<0.05), whereas MRP-1 expression was significantly linked with tumor size, depth of wall invasion, lymph node metastasis, TNM stages and differentiation status (P<0.05). ATG-5 expression was positively correlated with MRP-1 (rp = 0.616, P<0.01). Increased expression of ATG-5 and MPR-1 was significantly correlated with poor overall survival (OS; P<0.01) and disease free survival (DFS; P<0.01) of our GC cohort. Furthermore, we demonstrated that ATG-5 was involved in drug resistant of GC cells, which was mainly through regulating autophagy. Our data suggest that upregulated expression of ATG-5, an important molecular feature of protective autophagy, is associated with chemoresistance in GC. Expression of ATG-5 and MRP-1 may be independent prognostic markers for GC treatment.

## Introduction

Despite a considerable decline in its incidence rate in many developed countries, gastric cancer (GC) remains the fourth most commonly diagnosed malignancy, and the second leading cause of cancer-related deaths worldwide [1]. Over the past decades, standard multimodal treatment strategies together with other recommended options (e.g. D2 dissection and adjuvant chemotherapy) have failed to cure a large proportion of patients affected with GC, especially for those with advanced and metastatic diseases, with worse survival rates being seen probably due to the presence of chemoresistance during treatment [2]. Hence, identification of novel molecular events underlying the development of this malignancy and its poor prognosis as well as understanding the mechanisms of GC chemoresistance are urgently required for more effective clinical intervention and better management of patients.

Under physiological conditions, autophagy is a lysosome-dependent self-digesting system primarily responsible for removal and recycling of long-lived proteins and damaged/obsolete intracellularorganelles in order to maintain cell homeostasis [3]. The proteins and organelles destined for destruction are sequestered within “double-membrane” vacuoles (autophagosomes), followed by fusion with lysosomes to build complexes known as autophagosomes, where the contents are degraded by lysosomal hydrolases [4]. It has been documented that autophagy could be induced in response to many unfavorable conditions including nutrient deprivation, oxidative stress or DNA damages and serves as an adaptive cell mechanism, eventually allowing cells to survive and proliferate, while extensive or persistent autophagy results in cell death [5]. Impairments in physiological activation, assembly and function of the autophagic pathway have been increasingly observed in a wide variety of human cancers, although the exact role played by autophagy in cancer genesis and progression is still under controversy. Some data favor the idea that autophagy suppresses tumorigenesis, whereas other evidence suggest that autophagy is able to trigger tumor initiation and protects tumor cells from undergoing apoptosis [6]. Interestingly, inhibition of autophagy was recently found to enhance the anti-tumor activity of several cytotoxic agents. Li and colleagues reported that autophagy was activated as a protective mechanism against the cellular effects of 5-FU-treatment and inhibition of autophagy by 3-methyladenine augmented 5-FU-induced apoptosis in colon cancer cells [7], [8]. On the other hand, some anticancer drugs (e.g. cetuximab and dasatinib) were demonstrated to induce autophagic cell death through different mechanisms in some cancer cells [9]–[13]. The molecular machinery by which autophagy regulates survival or death of neoplastic cells remains largely obscure hitherto. The autophagy pathway is a highly-modulated dynamic process predominantly executed by the autophagy-related (ATG) family of genes, which is governed by several key kinases including mTOR, PI3k/Akt, AMPK and MAPK [14], [15]. ATG-5 is a central regulator necessary for autophagy in terms of its involvement in autophagosome elongation [16]. Enforced expression of ATG-5 sensitized tumor cells to anticancer drug treatment both *in vitro* and *in vivo*; in contrast siRNA-mediated inhibition of ATG-5 led to partial resistance to chemotherapy [17].

Postoperative adjuvant chemotherapy is presently a major treatment for GC; however, the overall efficacy of chemotherapy remains poor possibly as a consequence of the presence of multi-drug resistance (MDR) phenotype. Unlike other tumor entities, expression of the classical MDR-mediating molecules such as glutathione S-transferase and multidrug resistance gene 1 is not very prevalent in GC tissues, indicating that there might exist a complicated mechanism for the development of MDR in this malignant disease [18]. As one of the classical drug-resistant mechanisms, multidrug resistance-associated protein 1 (MRP1/ABCC1) has been found to be strongly expressed in GC and thus may exert pivotal roles in mediating MDR in GC [19], [20]. However, it remains unknown whether MRP-1 expression is associated with ATG-5 expression. And also whether autophagy is involved in chemoresistence in GC patients is unclear.

In the present study, we first employed immunohistochemistry to investigate the expression profile of ATG-5 and MRP1 in a sum of 135 GC patients who received ECF (epirubicin, cisplatin and 5-FU) adjuvant chemotherapy following surgical resection. The correlations between ATG-5 and MRP-1 expression as well as their expression with various clinicopathological features of GC and clinical outcomes were also assessed.

## Materials and Methods

### Patients and tissue samples

A total of 135 GC patients consisting of 91 males and 44 females who underwent surgery at the Department of Gastrointestinal Surgery, Xiangya Hospital, Central South University (C.S.U), China, between January 1st 2007 and December 31st 2008 were enrolled in this study. The average age of the cohort was 53.62±9.73 years, with a range of 26 to 72. Theprimary GC tumor tissuesand matched non-cancerous(NC) tissues located at least 5 cm away from the tumor core were obtained after surgical resection and immediately processed and stored until further use. None of the recruited patients had chemotherapy or radiotherapy prior to surgical operation. The histopathological diagnosis was carried out preoperatively and confirmed by surgery. All participants with stage IB to IV tumors received ECF chemotherapy after surgery (Dose: epirubicin 50 mg/m^2^ on day 1, cisplatin 60 mg/m^2^ on day 1 and continuous intravenous infusion of 5-FU 500 mg/m^2^/d for 4 days, repeated every 3 weeks up to 24 weeks). The clinical characteristics of these patients were listed in Table 1.

**Table 1:** Association between ATG-5 expression and clinicopathological characteristics of GC patients.

Factors	Patients (n = 135)	ATG-5 expression (%)				*P*
		0–99	100–199	200–299	300–400	
Gender						0.99
Male	91	21.98	14.29	46.15	17.58	
Female	44	22.73	15.91	45.45	15.91	
Age (years) Mean±SD	135	56.00±9.09	54.5±10.52	53.05±9.13	51.48±11.17	0.370▴
Tumor size (cm)						0.15
<5.0	80	25	18.75	43.75	12.5	
≥5.0	55	18.18	9.09	49.09	23.64	
Location of tumor						0.086
upper	21	19.05	33.33	38.1	9.52	
middle	35	11.43	12.5	54.29	22.86	
low	79	27.85	11.39	44.3	16.46	
H. pylori infection						0.961
Positive	73	23.29	15.07	43.84	17.81	
Negative	62	20.97	14.52	48.39	16.13	
Depth of wall invasion						<0.001
T1	10	50	20	30	0	
T2	15	66.67	13.33	13.33	6.67	
T3	32	25	12.5	43.75	18.75	
T4	78	8.97	15.38	55.13	20.51	
Lymph node metastasis						0.056
N0	18	22.22	33.33	38.89	5.56	
N1	36	33.33	5.56	50	11.11	
N2	44	22.73	9.09	47.73	20.45	
N3	37	10.81	21.62	43.24	24.32	
Distant metastasis						0.018
M0	131	22.9	15.27	46.56	15.27	
M1	4	0	0	25	75	
TNM stages						<0.001
IB	10	70	10	20	0	
II	31	45.16	32.26	22.58	0	
III	90	10	10	57.78	22.22	
IV	4	0	0	25	75	
Differentiation status						0.412
Well	17	41.18	17.65	23.53	17.65	
Moderate	60	20	16.67	48.33	15	
Poor	58	18.97	12.07	50	18.97	

▴ Statistical analyses were carried out with the One-Way ANOVA test and others were carried out with the Pearson’s χ^2^ test.

All cases in this study were reviewed and all specimens were histopathologically re-examined in October, 2012. The depth of wall invasion, regional lymph node metastasis, and histological grade were confirmed by the same group of two experienced senior pathologists. The patients were categorized based on the differentiation status of cancer cells into three histological grades: well, moderate and poor. Based on a combination of loco-regional tumor involvement and the presence of metastasis, all cases were staged according to the TNM Classification of Malignant Tumours (TNM) stage grouping [21]. For the analysis of survival, the date of operation was used to represent the start point of the follow-up visit. Patients who died of other diseases rather than GC or other unexpected events were excluded from the case collection. The cause of death recruited in this study was aggravation of GC. The overall survival (OS) was calculated as a period starting from the date of the initial surgery to the date of death, or the date of the last follow-up as the end point. The disease free survival (DFS) was defined as the time interval from surgery until the date of local relapse or first distant organ metastasis. Informed written consent was obtained from each patient before surgery and this study was approved by the Research Ethics Committee of Central South University, China. All specimens were handled and made anonymous according to the ethical and legal guidelines.

### Immunohistochemistry

The fresh specimens were fixed in 10% neutral buffered formalin and subsequently embedded with paraffin. The paraffin-embedded tissues were cut at 4 µm and then deparaffinized with xylene and rehydrated for further H&E or peroxidase immunohistochemistry staining by using the DAKO EnVision System. In brief, following proteolytic digestion and blocking with endogenous peroxidase, tissue slides were incubated with the primary antibodies (ATG5: ab54033; MRP1: ab32574; Abcam Inc., Cambridge, UK) against respective target proteins at a dilution of 1∶500 overnight at 4°C. After washing with PBS, peroxidase labeled polymer and substrate-chromogen were then employed in order to visualize the immnohistochemical staining. Finally, sections were counterstained with hematoxylin, cover-slipped with mounting medium, and examined by light microscopy. All the procedures were performed at the Department of Pathology, Xiangya Hospital, C.S.U. Slides were interpreted independently by two experienced pathologists, who were blind to patients’ information. We quantified staining intensity and percentage of stained cells using a previously described approach [22], [23]: the percentage of positively stained cells (0%–100%) was multiplied by the dominant intensity pattern of staining, considering 1 as negative or trace, 2 as weak, 3 as moderate and 4 as strong. Therefore, the overall score ranged from 0 to 400. Patients were subsequently categorized into four different subgroups: score 0–99, score 100–199, score 200–299 and score 300–400.

### Western blot analysis

Whole cell extracts were prepared using 0.14 M NaCl, 0.2 M triethanolamine, 0.2% sodium deoxycholate, 0.5% Nonidet P-40 and supplemented with a protease inhibitor (all of the products were from Sigma, St. Louis, Missouri, USA). Then, protein sample was run through a 12% sodium dodecyl sulfate-polyacrylamide gel electrophoresis (SDS-PAGE) gel and transferred to a membrane. The transferred membranes were subsequently incubated overnight at 4°C with a primary antibody. After washing, the membrane was incubated with a horseradish peroxidase (HRP)-linked secondary antibody for 1 h at room temperature. The primary antibodies were anti-ATG-5 (Santa Cruz, CA, USA), anti-LC3A/B (abcam, Cambridge, UK) and anti- β-Actin (Santa Cruz, CA, USA). All reported results are the average ratios of three different independent experiments.

### Cell proliferation assay

Cells were seeded onto 96 well plates (10000 cells/well) for 24 h before treatment. MTT assays were used to assess cell proliferation at different time point after treatment. The MTT assay was performed as follows: MTT was added to each well and the plates were incubated at 37°C for 4 h. The MTT medium mixture was then removed and 150 µL of dimethyl sulfoxide (DMSO) was added to each well. The absorbance was measured at 570 nm using a multiwall spectrophotometer.

### RNA interference

siRNA duplexes targeting ATG-5 were synthesized as follows: siRNA-ATG5-486: GACGUUG GUAACUGACAAATT; siRNA-ATG5-695: GUCCAUCUAAGGAUGCAAUTT and siRNA-ATG5-938: GACCUUUCAUUCAGAAGCUTT. siRNA duplexes containing non-specific sequences were used as a negative control (NC): UUCUCCGAACGUGUCACGUTT. Different siRNAs were transfected separately into cells using the Lipofectamine 2000 reagent, and the medium was replaced 6 h after transfection.

### Real-time RT-PCR

Total RNA from the cell lines and tissues were extracted using the Trizol reagent (Invitrogen, Carlsbad, USA), following the manufacturer’s instructions. The concentration of RNA was measured using a spectrophotometer. A cDNA pool was synthesized using 1 µg of total RNA and TaqMan Reverse Transcription Reagents (Applied Biosystems, Foster City, USA) as described by the manufacturer. The expression of the target gene was evaluated using a relative quantification approach (2^−ΔΔCt^ method) with β-actin as the internal reference.

### Immunofluorescence assay

Cells were permeabilized with 0.3% Triton X-100 for 10 min followed by fixation with 2–4% Methanal for 15 min, and blocked with 3% sheep serum at room temperature for 60 min. Then, probed with primary antibodies anti-LC3B (Santa Cruz, CA, USA) overnight at room temperature, and cells were washed three times with PBS. Stained with Alexa Fluor 488 conjugated 488 rabbit anti-goat IgG for 1 h at room temperature, and then the cells were washed three times with PBS. Nuclei were visualized by staining with DAPI (Sigma, USA) for 2 min. The stained cells were observed with inverted fluorescence microscope.

### Statistical analysis

All statistical analyses were performed with the SPSS software package 15.0 for Windows (SPSS Inc., Chicago, IL, USA). Quantitative data were presented as Mean ± SD. Pearson’s χ^2^ test was used to compare the difference among ranked data, while one-Way-ANOVA test was carried out to compare the difference among quantitative data. Survival analyses were carried out by using the Kaplan-Meier method and compared by the log-rank test. The Cox-regression model was performed to evaluate the independent hazard ratio of each variable in the multivariate analysis. The correlation between ATG5 and MRP1 expression was examined using Bivariate Correlation (Pearson) test. Differences were considered as statistically significant when *P* values were less than 0.05.

## Results

### Expression of ATG-5 and MRP-1 in GC

**Figure 1:**
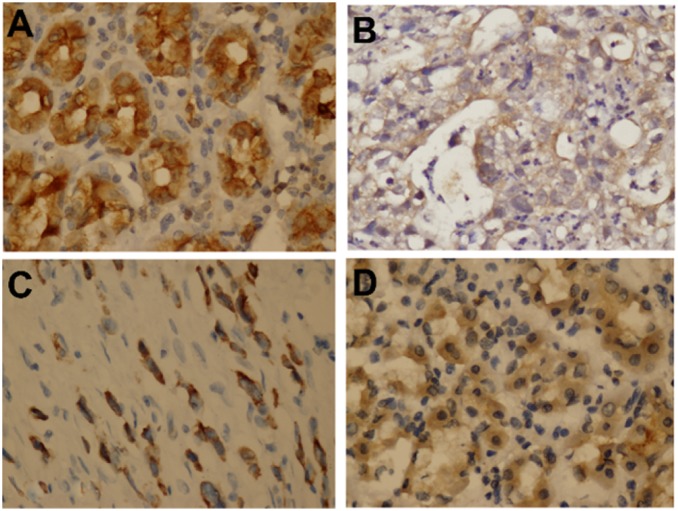
Representative images showing immunohistochemical staining for ATG-5 in non-tumorous and GC tumor tissues. (A) ATG-5 staining in non-cancerous gastric tissues scored 285(×200); (B) ATG-5 staining in GC tumor tissues scored 50(×400); (C) ATG-5 staining in GC tumor tissues scored 270(×400); (D) ATG-5 staining in GC tumor tissues scored 400(×200).

The expression pattern and location of ATG-5 and MRP-1 in our GC patients, who were treated with epirubicin, cisplatin and 5-FU adjuvant chemotherapy (ECF) following surgical resection, were examined using immunohistochemical analysis. Among the 135 GC specimens, 105(77.78%) were positive for ATG-5 immunoreactivity, and 107 (79.26%) were MRP-1 positive. As depicted in Figure 1, we found that ATG5 was predominantly expressed in the cytoplasm. Moreover, over-expression of ATG-5 was positively correlated with that of MRP-1 in GC. (r = 0.616, *P*<0.001), as revealed by the Bivariate Correlation test. The positive expression in adjacent non-cancerous tissues were 113(83.70%) for ATG-5 and 89(65.93%) for MRP-1. The data showed that both ATG-5 and MRP-1 were positively expressed in cancer and non-cancerous tissues, which suggest that ATG-5 and MRP-1 may be induced by chemotherapy in both tumor and non-tumor tissues. As all our patient samples were treated with ECF chemotherapy, and we found that both ATG-5 and MRP-1 were highly expressed and positively correlated in those samples. Meanwhile, previous study indicates that MRP-1 maybe associated with multi-drug resistance in GC. Together with previous finding, our results suggest that ATG-5 and MRP-1 may be involved in chemoresistance in GC patients.

### Associations between expression of ATG-5 or MRP-1 and clinicopathological characteristics of GC

**Figure 2:**
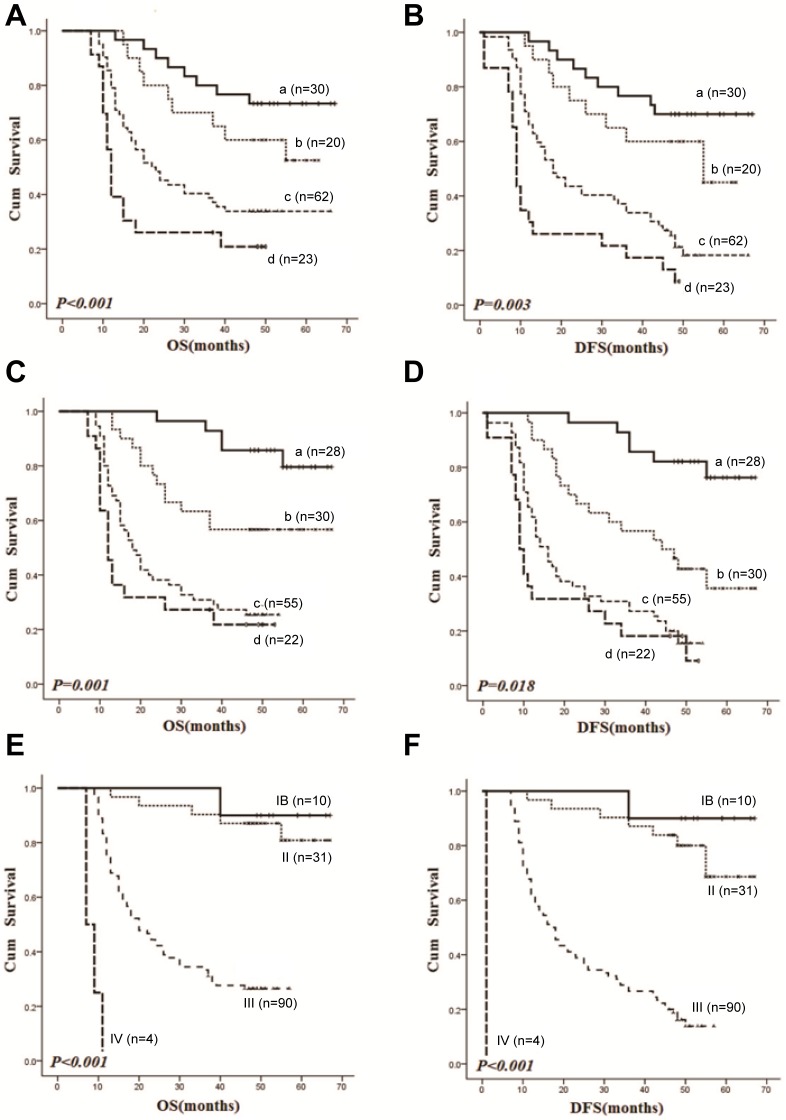
Kaplane-Meier statistical analyses showing OS and DFS in different subgroups of GC patients. (A and B) Patients were divided into four following subgroups based on ATG-5 immunostaining: scored 0–99 (curve a), scored 100–199 (curve b), scored 200–299 (curve c) and scored 300–400 (curve d). The difference among different subgroups was statistically significant as evaluated by overall Log-rank comparisons (OS: *P*<0.001, DFS: *P* = 0.003). Pairwise Log-rank comparisons showed that the subgroup D exhibited the poorest survival rates as compared to other subgroups (OS: *P*<0.05, DFS: *P*<0.01). (C and D) Patients were divided into four following subgroups based on MRP-1 immunostaining: scored 0–99 (curve a), scored 100–199 (curve b), scored 200–299 (curve c) and scored 300–400 (curve d). The difference among various subgroups was statistically significant by overall Log-rank comparisons (OS: *P* = 0.001, DFS: *P* = 0.018). Pairwise Log-rank comparisons showed that the subgroup A had the most favorable prognosis among the four subgroups (OS: *P*<0.05, DFS: *P*<0.001). (E and F) Patients were divided into four subgroups according to different TNM stages. The difference among different subgroups was statistically significant by overall Log-rank comparisons (OS: *P*<0.001, DFS: *P*<0.001). Pairwise Log-rank comparisons showed that subgroups III or IV exhibited poorer survival rates than that of subgroups IB and II (OS: *P*<0.01, DFS: *P*<0.001).

The associations of ATG-5 and MRP1 expression with various clinicopathological parameters of GC are shown in Table 1 and Table 2, respectively. Expression of ATG-5 was significantly associated with depth of wall invasion, distant metastasis and TNM stages of GC (*P*<0.001, *P* = 0.018, *P*<0.001 respectively). MRP-1 expression was significantly associated with increased tumor size, depth of wall invasion, regional lymph nodes metastasis, TNM stages (*P* = 0.032, *P*<0.001, P = 0.016, P<0.001 respectively) and differentiation status (*P* = 0.005). To further determine the involvement o f ATG-5 and MRP-1 in the GC development, we performed survival analysis within our patient samples. Our survival analyses demonstrated that the total overall survival (OS) rate of our GC cohort was 43.70% with a mean survival of 39.849 months (95% CI, 35.636–44.061 months); whereas the disease free survival (DFS) rate was 34.07% with a mean survival of 35.802 months (95% CI, 31.618–39.986 months). We next classified the patients into four different subgroups according to the scores of immunohistochemistry staining. The Kaplan-Meier survival analysis revealed a higher ATG-5 expression was significantly associated with poorer OS (*P*<0.001) and DFS (*P* = 0.003). Pairwise comparisons indicated that patients carrying the highest ATG-5 expression (scores 300–400) had the poorest survival rates as compared to that of other subgroups (Figure 2A and 2B). Consistently, upregulated MRP-1 expression was found to be significantly associated with poor OS (*P* = 0.001) and DFS (*P* = 0.018) of our GC patients. The subgroup with the highest MRP1 expression scores (0–99) appeared to have the worst prognosis (Figure 2C and 2D) in comparison with other subgroups. Our data also showed that there was a significant correlation between TNM stages and survival of GC patients. Patients with stage III and IV tumors displayed poorer prognosis as compared to those harboring stage IB and II tumors (*P<0.01*) (Figure 2E and 2F). More interestingly, the Cox’s multivariate hazard regression model demonstrated that ATG-5 and MRP-1 expression levels and TNM stages were all independent and significant prognostic indicators for predicting the OS (*P* = 0.037, *P* = 0.005, *P*<0.001 respectively) and DFS (*P* = 0.004, *P* = 0.008, *P*<0.001 respectively) of GC (Table 3). Our data indicated the ATG-5 and MRP-1 were closely related with the GC development and may serve as poor prognosis markers in GC treatment.

**Table 2:** Association between MRP-1 expression and clinicopathological characteristics of GC patients.

Factors	Patients (n = 135)	ATG-5 expression (%)				*P*
		0–99	100–199	200–299	300–400	
Gender						0.675
Male	91	19.78	21.98	43.96	14.29	
Female	44	22.73	22.73	34.09	20.45	
Age (years) Mean±SD	135					0.084▴
Tumor size (cm)		54.61±10.31	56.23±8.97	53.31±9.56	49.45±9.61	0.032
<5.0	80					
≥5.0	55	22.5	30	33.75	13.75	
Location of tumor		18.18	10.91	50.91	20	0.15
upper	21					
middle	35	19.05	28.57	38.1	14.29	
low	79	22.86	11.43	40	25.71	
H. pylori infection		20.25	25.32	41.77	12.66	0.51
Positive	73					
Negative	62	16.44	23.29	41.1	19.18	
Depth of wall invasion		25.81	20.97	40.32	12.9	<0.001
T1	10					
T2	15	60	20	20	0	
T3	32	53.33	26.67	20	0	
T4	78	28.13	15.63	37.5	18.75	
Lymph node metastasis		6.41	24.36	48.72	20.51	0.016
N0	18					
N1	36	22.22	44.44	33.33	0	
N2	44	33.33	16.67	36.11	13.89	
N3	37	18.18	22.73	47.73	11.36	
Distant metastasis		10.81	16.22	40.54	32.43	0.193
M0	131	21.37	22.9	40.46	15.27	
M1	4	0	0	50	50	
TNM stages						<0.001
IB	10	70	20	10	0	
II	31	54.84	35.48	9.68	0	
III	90	4.44	18.89	54.44	22.22	
IV	4					
Differentiation status		0	0	50	50	0.005
Well	17	52.94	17.65	23.53	5.88	
Moderate	60	16.67	31.67	36.67	15	
Poor	58	15.52	13.79	50	20.69	

▴ Statistical analyses were carried out with the One-Way ANOVA test and others were carried out with the Pearson’s χ^2^ test.

**Table 3:** The multivariate Cox proportional hazard analysis of prognostic factors for OS and DFS rates of 135 GC patients.

Factors	OS				DFS			
	β	Wald	RR# (95.0% CI)	P	β	Wald	RR# (95.0% CI)	P
MRP-1(300–400)		13.035		0.005		11.843		0.008
MRP-1(0–99)	−1.797	9.388	0.166(0.052–0.523)	0.002	−1.785	10.408	0.168(0.0576–0.496)	0.001
MRP-1(100–199)	−1.121	7.799	0.326(0.148–0.716)	0.007	−0.938	6.578	0.391(0.191–0.802)	0.011
MRP-1(200–299)	−0.353	1.373	0.702(0.389–1.268)	0.216	−0.413	2.076	0.662(0.377–1.161)	0.146
ATG-5	0.379	5.538	1.460(1.065–2.002)	0.037	0.486	9.73	1.626 (1.198–2.207)	0.004
TNM stage	1.869	17.151	6.484(2.677–15.705)	<0.001	1.85	18.785	6.362(2.755–14.688)	<0.001
Tumor size	0.109	0.178	0.897(0.542–1.486)	0.673	0.091	0.146	0.913(0.573–1.456)	0.702
Differentiation status	0.177	0.821	1.194(0.814–1.752)	0.365	0.192	1.141	1.212(0.852–1.726)	0.285
Hp infection	−0.115	0.238	0.891(0.560–1.417)	0.626	−0.122	0.307	0.885(0.574–1.364)	0.579
Location	−0.021	0.017	0.979(0.710–1.350)	0.898	−0.091	0.356	0.913(0.677–1.231)	0.551
Gender	0.108	0.44	0.897(0.652–1.236)	0.507	0.187	0.579	0.829(0.512–1.343)	0.447

#Relative Risk; CI: confidence interval.

### ATG-5 was significantly upregulated in chemoresistant cells

**Figure 3:**
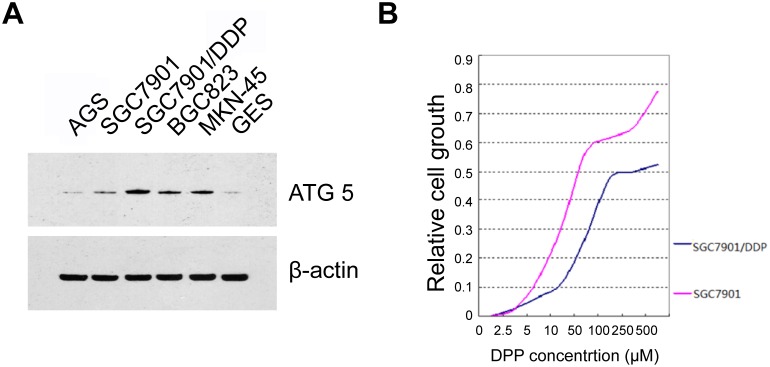
ATG-5 was upregulated in chemoresistant cells. (A) The level of ATG5 was detected in cell lines using western blot analysis. β -actin was used as internal controls.(B) The IC 25, IC50 and IC75 of SGC-7901 and SGC-7901/DDP cells were tested using the MTT assays after DPP treatment.

To further explore the role of ATG-5 in the tumorigenesis and drug resistant. We detected the protein expression in several gastric cancer cell lines (AGS, BGC-832, SGC7901, SGC7901/DPP and MKN45) and in an immortalized human gastric epithelial mucosa cell line (GES). Interestingly, we found that ATG-5 was dramatically overexpressed in DPP resistant cell line, SGC7901/DPP cells, compared with all the other cell lines which include DPP sensitive SGC7901 cells (Figure 3A). We further confirmed that SGC7901/DPP cells are resistant to DPP treatment. The IC 25, IC50 and IC75 were 15.4 µM, 38.7 µM and 93.53 µM in SGC7901 cells. In contrast, The IC 25, IC50 and IC75 were 120.03 µM, 271.9 µM and 423.7 µM in SGC7901/DPP (Figure 3B). It is about 5 to 9 times higher than that in non-drug resistant cells. Our finding strongly suggests that ATG-5 contributes to drug resistant of the GC cells.

### Inhibition of ATG-5 sensitized chemoresistant cells to drug treatment

**Figure 4:**
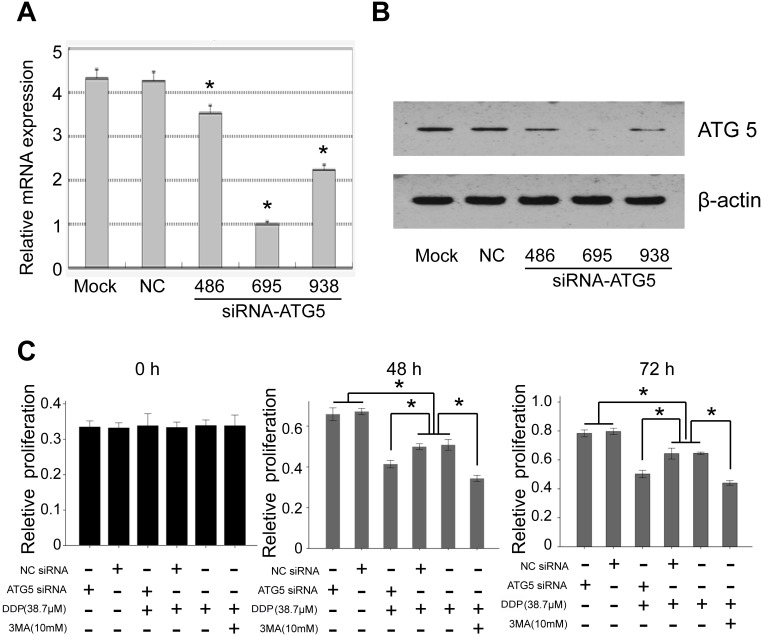
Silencing ATG-5 sensitized chemoresistant cells to drug treatment. (A) The mRNA level of ATG-5 was detected by real time PCR after treatment with siRNAs. GAPDH was used as internal controls. (B) The protein level of ATG-5 was detected by western blot after treatment with siRNAs. β -actin was used as internal controls. (C) The proliferation ability was tested using MTT assay 48 hours or 72 hours after different treatment.

To further prove that ATG-5 contributes to the drug resistant of the GC cells, we used small interfering RNAs (siRNAs) to knockdown the expression of ATG-5. Three siRNAs were designed. Our real time PCR and western blot results showed that all three siRNAs inhibited the expression of ATG-5 at both mRNA and protein level (Figure 4A and 4B). We chose one, siRNA-ATG5-695, with highest knockdown efficiency to perform the following experiment. We knockdown ATG-5 expression and then treated the cells with DPP. Cell proliferation ability was examined at 0, 48 and 72 hours after treatment. Our result showed that knockdowning ATG-5 did not affect cell proliferation in SGC7901/DPP cells compared with control siRNA (siRNA NC). DPP treatment alone slightly inhibited the proliferation of the cells. Interestingly, when we knockdown the expression of ATG-5 and treated the cells with DPP at the same time, the cell proliferation ability was further suppressed compared with cells treated with DPP alone 48 and 72 hours after treatment (Figure 4 C). Our data further support that ATG-5 contributes to the drug resistant of GC cells.

### Autophagy was involved in the drug resistant of DC cells

**Figure 5:**
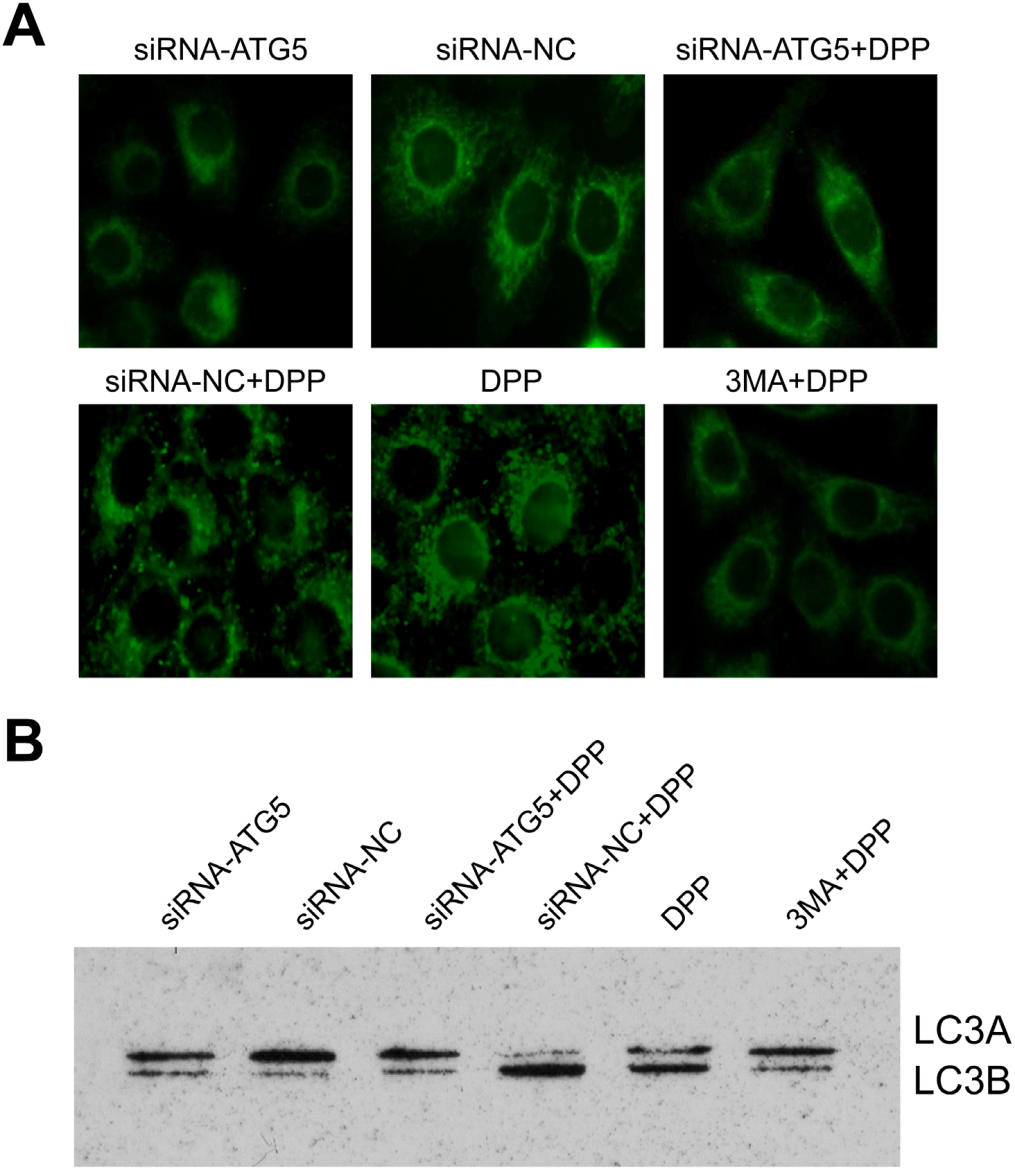
Autophagy was involved in the drug resistant of DC cells. (A) The autophagy was detected by immunofluorescence assay of LC3B in the cells 48 hours after different treatment. (B) The protein levels of LC3A and LC3B were tested by western blot. β-actin was used as internal controls.

As ATG-5 is a central regulator of autophagy, we speculated that autophagy may be involved in the drug resistant of GC cells. So we used 3MA, which is an autophagy inhibitor, to treat the drug resistant cells. As expected, we found that 3MA together with DPP treatment had a similar effect with ATG-5 kncokdown together with DPP treatment (Figure 4C and 4D). The data demonstrate that autophagy contributes to the drug resistant. Then, we examined whether autophagy was changed during the treatment. We used Immunofluorescence assay to detect LC3B expression level, which is an autophagy marker in the cells. Our data showed that autophagy was suppressed after silencing ATG-5 or treating the cells with 3MA (Figure 5A). And western blot result further confirmed that LC3A/B protein expression was only affected in cells treated with siRNA-ATG5 or 3MA. Accordingly, cell proliferation was further inhibited only when autophagy was inhibited (Figure 4 and figure 5). Therefore, our data revealed that ATG-5 was involved in the drug resistant of DC cells, which was mainly through affect the autophagy of the cancer cells.

## Discussion

GC remains one of the most frequent malignant tumors on a global basis in spite of its declining incidence and the total number is predicted to continuously climb as a result of population growth. In men, GC ranks the second in mortality rate; in women, it is the fourth in mortality [24], [25]. The crude mortality rate of GC in China was 25.2 per 100 000 [26]. In our study, we examined the expression of ATG-5 and MRP-1 in a cohort of GC patient after chemotherapy. Then, we demonstrated that ATG-5 was upregulated in cisplatin (DDP) resistant cell line. Furthermore, after ATG-5 expression or aotophogy was inhibited, the cancer cells were sensitized to DPP treatment. Our results provide new insight into the mechanism of chemoresistant in GC progression.

We evaluated the exression profile of ATG-5 and MRP-1 in 135 Chinese GC patients. In an agreement with previous report [22], our results showed that a high percentage of GC tissues expressed ATG-5, and ATG-5 expression was statistically associated with depth of wall invasion, distant metastasis and TNM stages of GC. These findings support a notion that high expression level of ATG5 may contribute to, some extent, a more aggressive and malignant phenotype in GC. This viewpoint is further supported by our finding of the association between higher ATG5 expression in GC and poorer prognosis of patients (see more discussion below). More importantly, we identified a positive correlation between ATG-5 and MRP1 expression in our GC cohort. Considering the fact that MRP1 is an ABC transmembrane transport protein well known to promote the MDR phenotype in GC, it is reasonable to propose that ATG-5 may be also implicated in conferring GC chemoresistance through certain unknown molecular mechanisms.

It is widely accepted that recurrence and metastasis are two major hurdles in our efforts to improve low OS and DFS survival rate of GC. Chemoresistance remains one of the most important reasons leading to tumor repopulation/recurrence following treatment. Appropriate option of individual treatment will be undoubtedly beneficial to improve the clinical outcome; nonetheless, current treatment decision is mostly dependent on the TNM stages [27], [28]. Our survival analyses in the 135 GC patients with stage IB to IV tumors revealed that both ATG-5 and MRP-1 expression were able to independently predict the OS and DFS after treatment with adjuvant ECF chemotherapy, suggesting that monitoring their expression levels in combination of conventional prognostic markers may provide us with additional valuable information for a better evaluation of chemotherapy effect in GC patients. Interestingly, we found the ATG-5 was overexpressed in drug resistant GC cell lines. And silencing ATG-5 can sensitized the drug resistant cells to chemotherapy again. Our data suggest that ATG-5 may be a target for chemoresistant paitents.

Accumulating evidence has suggested that autophagy is capable to trigger both cell survival and cell death under different contexts. Liu et al reported that through inhibition of the PI3K/Akt/mTOR pathway, β-elemene could induce protective autophagy to assist GC cells better adapt to stressful conditions and protect them from undergoing apoptosis death [29]. Furthermore, recent studies have shown that the PI3K/Akt/mTOR signaling pathway is frequently activated in human gastrointesti­nal malignancies [30]. The PI3K/Akt signaling also modulates MDR in GC cell through the regulation of p-glycoprotein, Bcl2 and Bax [31]. Likewise, some anticancer agents have been reported to inhibit mTOR signaling and induce autophagy in cancer cells by degrading many major components in the mTOR axis [14], [32]. Overall, these data suggest that autophagy could be induced during chemotherapy, and suppression of autophagic pathways using autophagy inhibitor have potential to improve the chemotherapeutic effectiveness in GC patients with ATG-5 high expression. In support, we found that when autophagy was inhibited, the drug resistant cells were also sensitized to drug treatment again as silencing ATG-5 expression. So, our result support that autophagy contributes to chemoresistant in patient.

In summary, over-expression of ATG-5, a key molecular player of the autophagic pathway, is associated with chemoresistance in GC. Expression of ATG-5 and MRP-1 could be considered as independent prognostic markers for predicting OS and DFS of GC patients based on the currently obtained data. On the basis of TNM stages, detection of their expression levels may be clinically meaningful for better prediction of chemotherapeutic treatment outcomes in patients suffering from this malignant disease. Future studies involving assessment of a larger number of cases, ideally from a different ethnic background, are definitely warranted to confirm our findings in this study.
